# The association between patients' preferred treatment after the use of a patient decision aid and their choice of eventual treatment

**DOI:** 10.1111/hex.13045

**Published:** 2020-03-13

**Authors:** Carmen S. S. Latenstein, Floris M. Thunnissen, Bastiaan J. M. Thomeer, Bob J. van Wely, Marjan J. Meinders, Glyn Elwyn, Philip R. de Reuver

**Affiliations:** ^1^ Department of Surgery Radboud University Medical Center Nijmegen The Netherlands; ^2^ Department of Surgery Bernhoven Uden The Netherlands; ^3^ Scientific Institute for Quality of Healthcare (IQ Healthcare) Radboud University Medical Center Nijmegen The Netherlands; ^4^ The Dartmouth Institute for Health Policy and Clinical Practice Dartmouth College Hanover NH USA

**Keywords:** general surgery, patient decision aid, patients' preference, shared decision making

## Abstract

**Objective:**

To investigate the association between patients' preferred treatment and eventual treatment. Second, to compare patients with surgical treatment to watchful waiting in order to identify predictive factors for surgery.

**Methods:**

A single‐centre retrospective study was performed between December 2015 and August 2018. Patients (≥18 years) who used a patient decision aid (PDA) for gallstones or inguinal hernia were included. After their first surgical consultation, patients received access to an online PDA. The patients' preferred treatment after the PDA was compared with their choice of eventual treatment. Multivariable regression analyses were performed for predictive factors for surgery.

**Results:**

In total, 567 patients with gallstones and 585 patients with an inguinal hernia were included. Of the patients with gallstones, 121 (21%) preferred watchful waiting, 367 (65%) preferred surgery, and 79 (14%) were not sure. The patients' preferred treatment was performed in 85.9%. Frequent pain attacks (OR 2.1, 95% CI 1.1‐3.9, *P* = .020) and preference for surgery (OR 4.4, 95% CI 1.9‐10.1, *P* = .001) independently predicted surgery. Of the patients with an inguinal hernia, 77 (13.2%) preferred watchful waiting, 452 (78.8%) preferred surgery, and 56 (9.6%) were not sure. The patients' preferred treatment was performed in 86.0%. The preference for surgery (OR 5.2, 95% CI 2.5‐10.6, *P* < .001) independently predicted surgery and worry about complications predicted avoidance of surgery (OR 0.5, 95% CI 0.2‐1.0, *P* = .037).

**Conclusion:**

This study, reflecting current clinical care, shows that patients' preferred treatment after using a PDA matches their eventual treatment choice in 86% of patients with gallstones or an inguinal hernia. In these patients, symptoms and patients' preference for surgery independently predicts eventual choice of surgery.

## 1 INTRODUCTION

An increasing number of patients want to be involved when a decision needs to be made between treatment options, commonly called shared decision making (SDM).^1^ As time is a limiting factor for SDM in the consulting room, SDM is frequently facilitated by (online) patient decision aids (PDAs). PDAs present comparative information about advantages and disadvantages of available options and evaluate personal values and preferences of the patient.^2^, ^3^, ^4^

PDAs are proven to be effective in improving knowledge, reducing decisional conflict and, moreover, changing patients' preferred treatment.^5^ Our group recently showed that PDAs reduce operation rates in patients with gallstones or an inguinal hernia.^6^ This change may be caused by the impact of the PDA on patients' preferences and cause a decision shift.^7^ Patients' preference on treatment is influenced by several factors. First, the preference is influenced by the patients' symptoms and their concern about the course of the condition. Second, the physician's advice is important in development of patients' preferred treatment.^8^, ^9^, ^10^ Third, treatment options, risks associated with each option and expectations of results can influence patients' preference.^7^, ^11^, ^12^ A PDA additionally informs a patient on treatment options and associated risks, but also explores patients' values to determine what is most important for the individual patient. Current PDAs consist of a personal value clarification exercise, which helps patients to identify their own values and find the treatment option most consistent with their preference. However, it is unknown which personal values are important in the preference for surgery.^13^ Knowledge concerning factors influencing patients' preferences and treatment decisions are important to improve SDM.^14^

Gallbladder surgery and hernia repair are both, to a degree, considered to be examples of preference‐sensitive care.^15^ International guidelines support watchful waiting in selected patients if symptoms are mild and the condition does not show signs of potential complications.^16^, ^17^, ^18^ Cholecystitis or biliary pancreatitis are potential complications of gallstones, while in case of an inguinal hernia, a complicated course may result in a bowel incarceration. Nevertheless, a non‐operative treatment is important to consider, especially since it is known that cholecystectomy is a sub‐optimal solution to treat pain in patients with gallstones and abdominal symptoms and the long‐term outcomes of hernia repair are variable.^19^, ^20^, ^21^ These variations in outcomes make it essential that patients are invited to participate in treatment decisions based on information about potential complications, surgical morbidity or the outcome after conservative management.

Current research mostly focuses on the patients' preferences, but information about the association between preferences and their choice of eventual treatment is not yet reported.^7^ Therefore, the aim of this study was to investigate the association between the preferred treatment option after PDA usage and the eventual choice of treatment. Secondly, we identified clinical factors and patient values that predicted a decision to have surgery.

## 2 METHODS

### 2.1 Study design and study population

A single‐centre retrospective study was performed in a community hospital in the Netherlands. Patients were eligible for participation in the study if they were ≥18 years of age and used a PDA between December 2015 and August 2018. All patients with an emergency indication for surgery were excluded.

PDAs for patients with gallstones or an inguinal hernia were integrated into the standard workflow at the surgical outpatient clinic in December 2015. Physicians were trained in SDM to support the implementation of PDAs. This training covered the use of the PDA with a patient. Moreover, a workgroup was formed, consisting of at least a medical doctor, a project manager (from the hospital), a nurse of the outpatient clinic and a department manager of the outpatient clinic. Objectives for the use of the PDAs were set, as well as the care process for patients. Workshops were organized to inspire about SDM, to explain how the PDAs work and to train communication skills to empower patients to participate in the decision‐making process, assess patients' preferences and health values.

**Figure 1:**
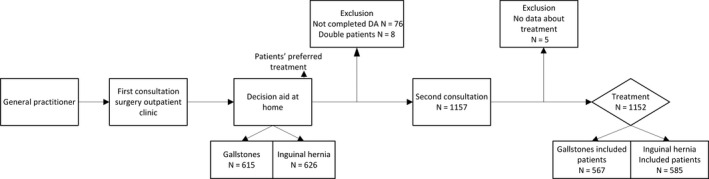
Flow chart of surgical consultations, decision aid and inclusion of patients. Flow chart of surgical consultations and the timing of the online decision aid and measurement of patients' preferred treatment. This flow chart also shows the inclusion of patients. 615 patients with gallstones used the decision aid and eventually 567 patients were included. Between December 2015 and August 2018 626 patients with an inguinal hernia used the decision aid and eventually 585 patients were included

In the Netherlands, patients are referred to the surgical outpatient clinic by their general practitioner after the diagnosis of symptomatic gallstones or after the diagnosis of an inguinal hernia, based on ultrasound imaging in the local hospital if applicable.^16^ During the first consultation in the outpatient clinic, the surgeon takes the history, performs physical examination and counsels patients. The condition and choices in treatment, including conservative treatment are explained. The risks of treatments are discussed after which, a PDA is introduced. A new appointment is made after 1‐2 weeks ensuring careful decision making and informed consent. During the second appointment, the results of the PDA are discussed with the patient and treatment choice is made using a SDM approach (Figure 1).

The local medical ethical committee and the boards of directors approved the study protocol (registration number 2018‐4587). This research was performed in accordance with the ethical standards of the updated Helsinki Declaration of 2013. The results are presented according to the Strengthening the Reporting of Observational studies in Epidemiology (STROBE) guidelines.^22^

### 2.2 Patient decision aids

The online PDAs (https://www.decisionaid.info) were developed by patients and physicians according to the International Patient Decision Aids Standards (IPDAS) (http://ipdas.ohri.ca/). Surgery and watchful waiting are compared in five steps (Supplementary file S1 and S2). Detailed information about the five steps of the PDAs have been described elsewhere.^6^

### 2.3 Outcome and data collection

To obtain information about the association between preference and eventual choice of treatment after PDAs, we compared the patients' preferred treatment after PDA and their eventual choice of treatment. The outcome of this study is the percentage of patients in which the patients' preferred treatment matched their eventual choice of treatment. Secondary outcomes were as follows: certainty about their preferred treatment and predictive factors (patient characteristics, patients' preference, disease burden and personal values) for surgery.

Data about patients' preferred treatment, certainty about their preference, disease burden and personal values were derived from the PDA. Questions about disease burden and personal values were presented as two statements on each side of a slider (ranging from −5 to 5). For every statement, the left side represented watchful waiting and the right side represented surgery. Patients moved the triangle to the side they agreed most with. Patients with gallstones specified their disease burden and personal values about *frequency of pain attacks, complaints* and *concerns about surgery*. Patients with an inguinal hernia specified their disease burden and personal values about *discomfort, concerns about surgery, bowel incarceration* and *surgical complications*. The patients were asked to point out their preferred treatment choice, on a slider ranging from −5 (watchful waiting) to 5 (surgery). Last, patients indicated how certain they were with their preferred option on a slider, ranging from 0 to 10. Age, sex and their eventual choice of treatment were derived from the hospital register in January 2018.

### 2.4 Statistical analysis

Patients who had not completed the PDA, whose data about their eventual choice of treatment were not available from the hospital register, and patients who had completed multiple PDAs for the same condition were excluded from the analysis. The data of patients with gallstones and inguinal hernia were analysed and presented separately.

The patients' preferred treatment, originally scored on an eleven‐point scale from −5 to 5, was first transformed into a categorical scale. Scores of −5 to −2 represented the preference watchful waiting; −1 to 1 represented not sure and 2 to 5 represented surgery. The sensitivity of this transformation was tested by also applying an alternative transformation: −5 to −3 represented watchful waiting; −2 to 2 represented not sure/intermediate; and 3 to 5 represented surgery (no differences with main outcome, data not shown). The value clarification exercise also consisted of 11‐point scales from −5 to 5; these were transformed with the same cut‐off scores.

Categorical data (sex, patients' preferred treatment, their eventual choice of treatment, disease burden, and personal values from the value clarification exercise) were summarized by frequencies and proportions. Continuous data (age, certainty) were presented as mean ± standard deviation (SD) when normally distributed and median with range or interquartile range (IQR) when non‐normally distributed. To investigate the association between patients' preferred treatment and their eventual choice of treatment, a bar chart with both variables was presented.

Patients were compared based on their eventual choice of treatment (watchful waiting and surgical) by age, sex, disease burden, personal values and their preferred treatment. Comparison of these variables was done using chi‐squared test for dichotomous data, Student's *t* test for normally distributed continuous data and the Mann‐Whitney *U* test for skewed continuous data. Testing for normality of data distributions was based on the Shapiro‐Wilks test.

To identify predictive factors for performing surgery, first univariate logistic regression analyses were performed with their eventual choice of treatment (watchful waiting/surgery) as dependent variable and patients' preferred treatment (watchful waiting, not sure and surgery), age, sex, disease burden and personal values, as independent variables. These were all included in the multivariable model. The outcomes of the univariable and multivariable analyses were presented as an odds ratio (OR) and 95% confidence interval (CI).

Associations with a *P*‐value <.05 were considered statistically significant. All missing values were considered to be at random and were excluded from analyses. Analyses were performed using SPSS statistics version 25.0 (IBM).

## 3 RESULTS

### 3.1 Patients

Between December 2015 and August 2018, 1075 patients were referred to the surgical outpatient clinic with symptomatic gallstones and 1219 patients with an inguinal hernia. PDAs were used by 615 patients (74.5%) with gallstones and 626 patients (76.1%) with an inguinal hernia. In addition, 89 patients (7.2%) were excluded from analyses, because patients had not finished the PDA, no data were available about eventual choice of treatment, or patients had completed multiple PDAs (Figure 1).

In total, 567 patients with gallstones (median age 52 years IQR 41‐63, 30.0% male) and 585 patients with an inguinal hernia (median age 60 years, IQR 49.5‐70.5, 92.1% male) were eligible for analysis. Baseline characteristics are described in Table 1.

**Table 1:** Characteristics and treatment of included patients

	Gallstones (n = 567)	Inguinal hernia (n = 585)
Age (median, IQR) years	52 (41‐63)	60 (49.5‐70.5)
Sex (M:F, %M)	170:396 (30.0)	539:46 (92.1)
Patients' treatment preference		
Watchful waiting (n, %)	121 (21.3)	77 (13.2)
Not sure (n, %)	79 (13.9)	56 (9.6)
Surgery (n, %)	367 (64.7)	452 (78.8)
Eventual choice of treatment		
Watchful waiting (n, %)	172 (30.3)	147 (25.1)
Surgery (n, %)	395 (69.7)	438 (74.9)

### 3.2 Patients' preferred vs eventual choice of treatment

The patients' preferred treatment was their eventual choice of treatment in 85.9% of patients with gallstones. In patients with an inguinal hernia, the patients' preferred treatment was their eventual choice of treatment in 86.0%. In both groups, 14% of patients were not treated according to their preference reported in the DA.

**Figure 2:**
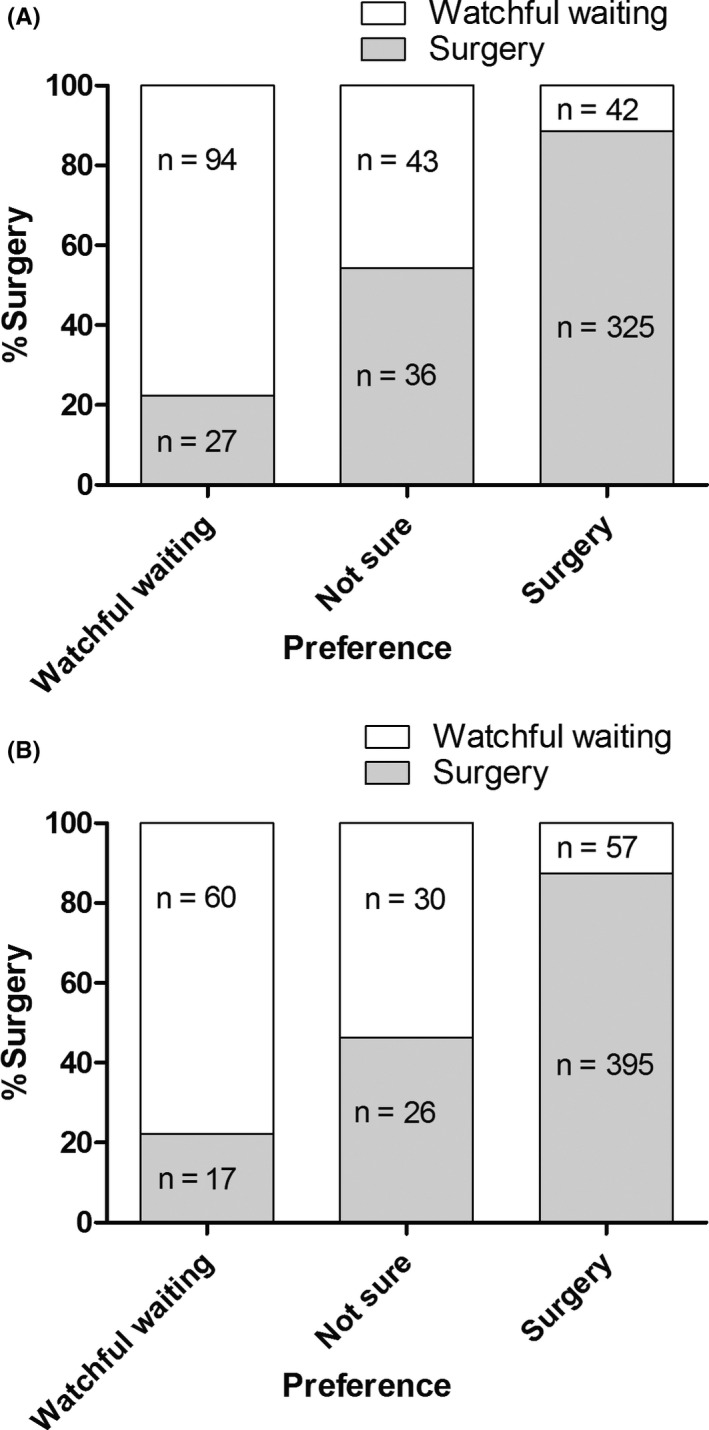
A, Gallstones—Eventual choice of treatment in patients with the preference watchful waiting, not sure and surgery. B, Inguinal hernia—Eventual choice of treatment in patients with the preference watchful waiting, not sure and surgery. Patients are divided by their preferred treatment. On the *y*‐axis, the percentage of eventually chosen surgeries is shown. The grey parts of the columns represent surgery, and the white parts represent watchful waiting

Of the patients with gallstones, 121 (21.3%) preferred watchful waiting at the end of the DA, 79 (13.9%) were not sure, and 367 (64.7%) preferred surgery (Table 1). In total, 395 patients (69.7%) underwent a gallbladder removal. Figure 2A shows the association between the patients' preferred treatment at the end of the PDA and their eventual choice of treatment.

Of the patients with an inguinal hernia, 77 (13.2%) preferred watchful waiting, 56 of patients (9.6%) were not sure, and 452 (78.8%) preferred surgery. In total, 147 patients (25.1%) were treated conservatively and 438 patients (74.9%) had surgery. Figure 2B shows the association between the patients' preferred treatment at the end of the PDA and their eventual choice of treatment.

### 3.3 Certainty about preferred treatment

For both conditions, patients who preferred surgery were more certain about their preference compared to patients who preferred watchful waiting. In patients with gallstones, median scores were 9.0 (IQR 8.0‐10.0) and 7.0 (IQR 5.5‐8.5), respectively, for the preference surgery and watchful waiting (*P* < .001). In patients with an inguinal hernia, median scores were 9.0 (IQR 8.0‐10.0) and 8.0 (IQR 7.0‐9.0), respectively, for surgery and watchful waiting (*P* < .001).

### 3.4 Predictive factors for surgery

In Tables 2 and 3, patients with a surgery were compared with patients who underwent a watchful waiting strategy. Patients with gallstones who underwent surgery were younger compared to patients who waited (50.1 ± 15.1 vs 55.6 ± 14.9, *P* < .001). Surgical patients were characterized by reporting more pain attacks, major complaints and less concern about surgery compared to patients with a watchful waiting policy (*P* < .001 for each comparison). In multivariable analyses, only frequent pain attacks (OR 2.1, 95% CI 1.1‐3.9, *P* = .020) and preference for surgery (OR 4.4, 95% CI 1.9‐10.1, *P* = .001) were independently predictive factors for surgery.

**Table 2:** Characteristics, disease burden, personal values and treatment preference of patients with gallstones with watchful waiting (n = 172) and surgery (n = 395)

	Watchful waiting (n = 172)	Surgery (n = 395)	*P*‐value	Univariable Odds Ratio (95% CI)	Multivariable Odds Ratio (95% CI)
Sex, male	47 (27.3)	123 (31.1)	.353	1.207 (0.811‐1.796)	1.425 (0.844‐2.406)
Age (median, IQR) years^a^	56.0 (45.5‐66.5)	50.0 (38‐62)	<.001	**0.967 (0.964‐0.988)**	0.992 (0.976‐1.008)
Pain attacks			<.001		
Infrequent pain attacks	74 (43.0)	42 (10.6)		**0.467 (0.268‐0.816)**	1.394 (0.656‐2.960)
Intermediate	42 (24.4)	51 (12.9)		1	1
Frequent pain attacks	56 (32.6)	302 (76.6)		**4.441 (2.699‐7.308)**	**2.118 (1.135‐3.952)**
Complaints			<.001		
Minor complaints	99 (57.6)	39 (9.9)		**0.210 (0.114‐0.386)**	0.565 (0.247‐1.295)
Intermediate	25 (14.5)	47 (11.9)		1	1
Major complaints	48 (27.9)	309 (78.2)		**3.424 (1.931‐6.071)**	0.903 (0.377‐2.164)
Worry about surgery			<.001		
Worried	83 (48.2)	76 (19.2)		**0.364 (0.214‐0.619)**	0.561 (0.284‐1.105)
Intermediate	29 (16.9)	73 (18.5)		1	1
Not worried	60 (34.9)	246 (62.3)		1.629 (0.974‐2.725)	0.814 (0.421‐1.575)
Treatment preference			<.001		
Watchful waiting	94 (54.7)	27 (6.8)		**0.240 (0.130‐0.445)**	**0.339 (0.163‐0.707)**
Not sure	36 (22.7)	43 (10.9)		1	1
Surgery	42 (24.4)	325 (82.3)		**6.647 (3.749‐11.20)**	**4.148 (1.791‐9.604)**

Note

Univariable and multivariable scores in bold have a *P*‐value < .05.

^a^ Analysed with Mann‐Whitney *U* test.

**Table 3:** Patient characteristics, disease burden, personal values and treatment preference of patients an inguinal hernia with watchful waiting (n = 147) and surgery (n = 438)

	Watchful waiting (n = 147)	Surgery (n = 438)	*P*‐value	Univariable	Multivariable
Sex, male (n, %)	131 (89.1)	408 (93.2)	.116	1.661 (0.878‐3.143)	1.515 (0.657‐3.492)
Age (median, IQR) years^a^	63.0 (53‐73)	58.0 (48‐68)	.002	**0.978 (0.965‐0.992)**	0.991 (0.975‐1.008)
Discomfort			<.001		
Little discomfort	83 (56.5)	64 (14.6)		**0.274 (0.512‐0.491)**	0.671 (0.320‐1.408)
Intermediate	22 (15.0)	62 (14.1)		1	1
Much discomfort	42 (28.6)	312 (71.2)		**2.636 (1.471‐4.724)**	1.879 (0.967‐3.651)
Worry about surgery			<.001		
Worried	34 (23.1)	37 (8.4)		0.544 (0.293‐1.009)	0.691 (0.300‐1.595)
Intermediate	35 (23.8)	70 (16.0)		1	1
Not worried	78 (53.1)	331 (75.6)		**2.122 (1.320‐3.411)**	1.167 (0.591‐2.305)
Worry about incarceration			<.001		
Not worried	63 (42.8)	73 (16.7)		**0.338 (0.205‐0.555)**	0.691 (0.347‐1.375)
Intermediate	37 (25.2)	127 (29.0)		1	1
Worried	47 (32.0)	238 (54.3)		1.475 (0.911‐2.388)	0.770 (0.413‐1.435)
Worry about complications of surgery			<.001		
Worried	42 (28.6)	40 (9.1)		**0.306 (0.173‐0.542)**	**0.467 (0.223‐0.975)**
Intermediate	37 (25.2)	115 (26.3)		1	1
Not worried	68 (46.3)	283 (64.6)		1.339 (0.848‐2.111)	0.936 (0.501‐1.748)
Treatment preference			<.001		
Watchful waiting	60 (40.8)	17 (3.9)		**0.327 (0.154‐0.694)**	0.589 (0.243‐1.428)
Not sure	30 (20.4)	26 (5.9)		1	1
Surgery	57 (38.8)	395 (90.2)		**7.996 (4.414‐14.48)**	**5.158 (2.508‐10.61)**

Note

Univariable and multivariable scores in bold have a *P*‐value < .05.

^a^ Analysed with Mann‐Whitney *U* test

In patients with an inguinal hernia, similar outcomes were found. Surgically treated patients were younger compared to patients who waited (56.8 ± 14.4 vs 61.1 ± 14.4, *P* = .002). Surgical patients were characterized by more discomfort, less concern about surgery, less concern about risks of surgery and more concern about bowel incarceration, compared to patients with watchful waiting (*P* < .001 for all comparisons). In multivariable analyses, the preference for surgery (OR 5.2, 95% CI 2.5‐10.6, *P* < .001) independently predicted surgery and worry about complications of surgery was negatively associated with surgery (OR 0.5, 95% CI 0.2‐1.0, *P* = .037).

## 4 DISCUSSION

The present study, reflecting real‐life clinical decision making, shows that general surgical patients' preference for either watchful waiting or surgery after a PDA is highly associated with their eventual choice of treatment. Patients who prefer surgery seem to be more certain about their preference compared to patients who prefer watchful waiting. In patients with gallstones, ‘frequent pain attacks' is an independent predictive factor for surgical treatment. In both patient groups, with gallstones or inguinal hernia, the patients' preference for surgery is an independent predictive factor for surgical treatment. In patients with an inguinal hernia, ‘worry about complications of surgery' is associated with avoidance of surgery. Fourteen percentage of patients are not treated according to their preference reported in the DA.

The majority of patients with gallstones or inguinal hernia prefer surgery after using a PDA. It could well be that patients' preference for surgery contributes to the nationwide operation rates in the Netherlands above 70% for both conditions.^19^ Despite the finding that watchful waiting is a good alternative in selected patients with symptomatic gallstones or an inguinal hernia,^16^, ^23^, ^24^ it looks like most patients prefer a quick and definitive solution for their symptoms.^25^, ^26^, ^27^, ^28^ That is in line with our finding that the majority of surgical patients report that they are very certain about their preference. This might be explained by the patients' anticipation on a surgical treatment after their general practitioner explained the potential cause of their symptoms and an ultrasound confirmed the diagnosis of gallstones or an inguinal hernia. SDM is in case of preference‐sensitive care even more important; counselling patients and create awareness of potential disadvantages of surgery is mandatory.

The way PDAs should be provided and integrated into the workflow is debated. Different PDA formats are available (booklet, audio, online), interactive or not, and PDAs can be provided at different locations or time points (at general practitioner, before surgical consultation, after first surgical consultation).^29^ In the present study, the online link to the PDAs was provided after the first consultation with the patients' surgeon. During the second consultation, the eventual choice of treatment was chosen. Alternatively, the PDA could be provided by the general practitioner, before referral to the surgeon. Implementation of PDAs and SDM earlier in the clinical pathway leads to the following advantages; improved patient knowledge, patients who are more clear about their values, reduced decisional conflict and potential; fewer referrals, reduced diagnostics and less invasive treatments.^5^, ^30^ Moreover, general practitioners are probably more aware of the patients' values due to a long‐lasting relationship and considerations and can alter expectations in a timely fashion.^31^

The discrepancy between patients' initially preferred treatment and different eventual choice of treatment is of interest. This study shows a discrepancy in 14% of patients with gallstones or inguinal hernia. This could reflect the surgeons' opinion who advised a different treatment, patients could have shifted their preference after usage of the PDA for example in conversation with family or friends, or the condition could have presented with signs of a potential complicated course (eg cholecystitis or bowel incarceration). It is of interest that patients who initially preferred watchful waiting less often received their preferred treatment compared to patients preferring surgery (77.8% vs 87.9%). Due to the retrospective aspect of this study, we were unable to analyse the potential causes of this phenomenon further.

This is the first study that shows the association between patients' preferred treatment, expressed after using a PDA, and their eventual choice of treatment. Strengthening the study is the real‐life study design, which shows daily clinical practice compared to controlled condition within randomized controlled trials. However, this study also comes with limitations. The major limitation of this study is missing information about the consultation with the general practitioner and the first surgical consultation. Available data are obtained from the PDAs, which were provided to patients after these two consultations. Therefore, we cannot assess the exact impact of the PDA on the patients' preferences, as we do not know their initial preferences. Secondly, due to the lack of a control group, we are not able to compare outcomes between patients with and without our intervention.

As this study only included patients with gallstones or an inguinal hernia who used a PDA, it remains unclear what happens with preferences of patients who consult their surgeon but do not use PDAs. It could be hypothesized that the discrepancy between the initial patients' preference and their eventual choice of treatment could be greater, because the increase in knowledge concerning treatment options and risks is missing. Finally, our research only assessed patient preferences in surgical candidates and the impact of PDAs in patients with non‐surgical conditions is of also of interest.

To overcome the methodological limitations of the present study, we started a prospective assessment (Dutch Trial Register NTR7501). As a result of longitudinal data collection, the influence of disease status, patients' values, preference shifts will clarify more clearly the discrepancy between patients' preferred treatment and their eventual choice of treatment.

To date, surgeons are mainly focused on potentially complicated course of a condition (eg cholecystitis and bowel incarceration) or surgical complications (eg bile duct injury or persistent pain), while patients are more focused on long‐term outcomes and working disability.^16^, ^23^, ^24^, ^32^ To improve SDM in clinical practice, physicians should be aware of this discrepancy.^33^ Furthermore, as our data show differences between surgically treated and non‐surgically treated patient, age and personal values, personalized patient DAs are the next step in improving SDM. The benefits and risks pointed out in the PDA, the option grid and the value clarification exercises should all be related to the characteristics of patients. Then, patients' preference can be better explored, and satisfaction of patients may increase.

In conclusion, patients reported preferred treatment after the use of a PDA is highly predictive for the eventual choice of treatment in patients with gallstones or an inguinal hernia. Fourteen percentage of patients with symptomatic gallstones or an inguinal hernia do not undergo preferred treatment. In patients with gallstones, their physical complaints are more predictive for a surgical intervention than their worry about surgery related complications.

## CONFLICT OF INTERESTS

All authors have completed the ICMJE uniform disclosure form at and declare the following: this research did not receive any specific grant from funding agencies in the public, commercial, or not‐for‐profit sectors. Glyn Elwyn has edited and published books that provide royalties on sales by the publishers: the books include *Shared Decision Making* (Oxford University Press) and *Groups* (Radcliffe Press). He has in the past provided consultancy for organizations, including the following: 1) Emmi Solutions LLC who developed patient decision support tools; 2) National Quality Forum on the certification of decision support tools; 3) Washington State Health Department on the certification of decision support tools; 4) SciMentum LLC, Amsterdam (workshops for shared decision making). He is the Founder and Director of &think LLC which owns the registered trademark for Option Grids™ patient decision aids. Founder and Director of SHARPNETWORK LLC, a provider of training for shared decision making. He provides advice in the domain of shared decision making and patient decision aids to: (a) Access Community Health Network, Chicago (Federally Qualified Medical Centers); (b) EBSCO Health Option Grids™ patient decision aids; (c) Bind Insurance; (d) PatientWisdom Inc; (e) abridge AI Inc Glyn Elwyn's academic interests are focused on shared decision making and coproduction. He owns copyright in measures of shared decision making and care integration, namely collaboRATE, integRATE, consideRATE, coopeRATE, toleRATE, Observer OPTION‐5 and Observer OPTION‐12. No other relationships or activities have influenced the submitted work.

## AUTHOR CONTRIBUTIONS

Latenstein and Thunnissen conceived and designed the study; acquired, analysed and interpreted data; and drafted the manuscript. Thomeer and van Wely acquired data and critically revised the article for important intellectual content. Meinders, Elwyn and de Reuver interpreted data and critically revised the article for important intellectual content. All authors gave final approval of the version to be published and agreed to be accountable for all aspects of the work.

## ETHICAL APPROVAL

The study was approved by the appropriate institution and/or national research ethics committee (registration number 2018‐4587).

## Supporting information

FileS1

FileS2

## Data Availability

The data that support the findings of this study are available from the corresponding author after reasonable request.
