# Supplementary material for: The association between patients' preferred treatment after the use of a patient decision aid and their choice of eventual treatment
Source: Health Expect. 2020 Mar 13;23(3):651–8. doi: 10.1111/hex.13045 (PMC7321744; doi:10.1111/hex.13045)
Supplement: Supplementary file 1 — FileS1 [file HEX-23-651-s001.pdf]

Welcome to the PATIENT+ decision aid

How can this decision aid help you?

There are various treatments available for people with gallbladder attacks. This decision aid provides you with the harms and benefits of each treatment. If you're unsure, your doctor can help you choose the best treatment for you.

At the end you can print this decision aid. This way you can go over it again and discuss everything with people you trust.

What is bile?

Bile is a fluid that helps your intestines in the digestion of fat. Bile is produced by the liver and stored in the gallbladder. The gallbladder is a small bag underneath the liver. If fat food needs to be digested, bile flows through a tube from the gallbladder to your intestine.

What are gallstones?

Gallstones are lumps of hardened bile (figure). They are formed in the gallbladder. It is not clear why it happens to some people and not to others. We know gallstones are more common:

- in women;
- during pregnancy
- in people who are obese.

Gallstones are common. But in most people gallstones don't cause any symptoms.

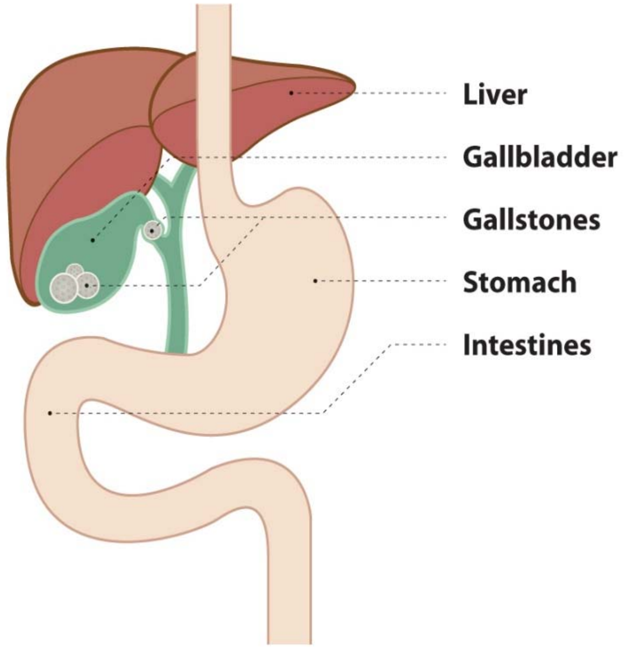

Figure: gallstones.

Gallbladder attack

Gallstones become symptomatic when they get stuck in the tube leading from the gallbladder to the intestine. This is called a gallbladder attack. When this happens people experience the following symptoms:

- severe abdominal pain on the right side just below the ribcage;
- sometimes they simultaneously experience back pain;
- nausea and vomiting;

An attack can last between half an hour and several hours.

Two out of three people get a second gallbladder attack.

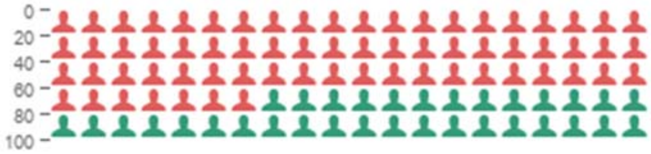

What are the treatment options?

There are two possible treatments:

**Watchful waiting:**	• During a gallbladder attack you take strong painkillers
**Surgery:**	• Removing the gallbladder

Watchful waiting

You can wait. If you get another attack, you can take painkillers. You can prevent another attack by living healthy and exercise. If you're overweight, try not to lose weight too rapidly; this increases the risk of gallbladder attacks.[1,2]

Benefits:

You do not have the risk of surgery. Maybe you are one out of three, who will never get an attack again.[7]

Risks:

If you wait, during a gallbladder attack, the gallbladder or pancreas could get infected. If that happens, you could get very sick for a long time. This happens to less than 1 out of 100 people.[3]

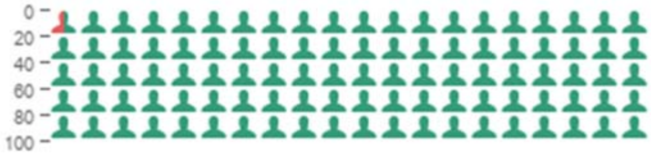

Surgery

During surgery, the gallbladder is removed. You can live without a gallbladder. Bile will go directly from the liver to the small intestine.

You'll be asleep during the surgery. During this surgery, the surgeon will make one incision beneath the bellybutton and three small incisions underneath the right side of the ribcage. The surgical instruments will be placed in the abdomen through these incisions (figure). With the

instruments the surgeon will remove the gallbladder. The surgery takes about one hour. Usually, you can go home the same day after surgery.

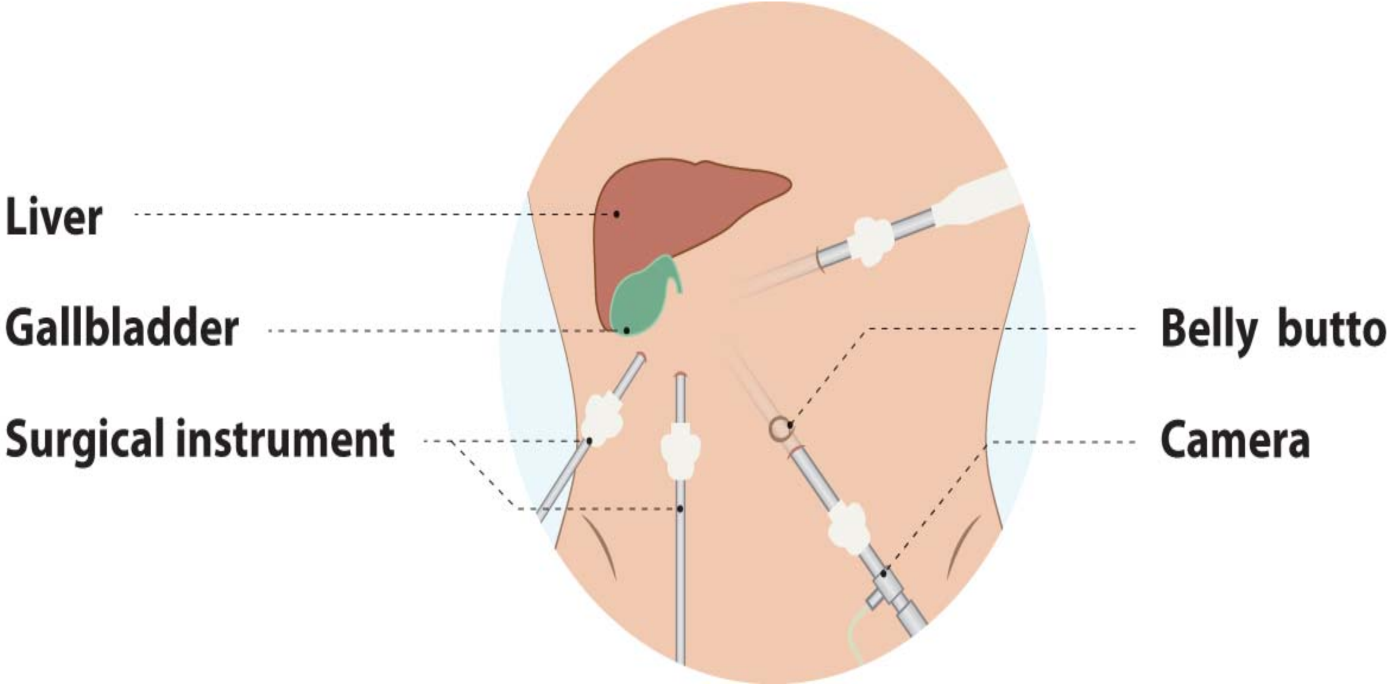

Figure: Surgical instruments to remove the gallbladder.

The days after surgery you’ll experience some abdominal pain. Yet, there are no limitations to your activities.

The risks of surgery

During or after surgery undesirable things can happen. We call this an adverse event. During the surgery to your gallbladder, the following can occur:[4, 5, 6, 8]

The wounds can become infected or you can bleed inside your abdomen. This happens to 1 out of every 100 patients.

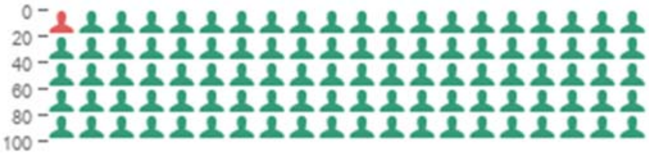

Your bile duct can get damaged. This occurs to 1 out of every 200 patients who have this surgery.

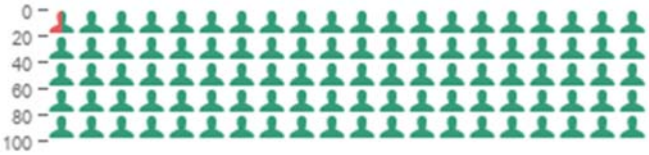

Also your small intestine could get damaged. This happens to 1 out of very 500 patients who undergo surgery. If this occurs, you will probably need a second surgery.

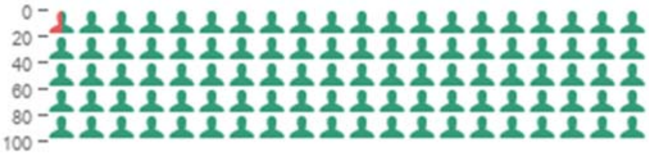

In 4 out of 100 people the incision will be extended during surgery (figure). If this is necessary recovery will take longer.

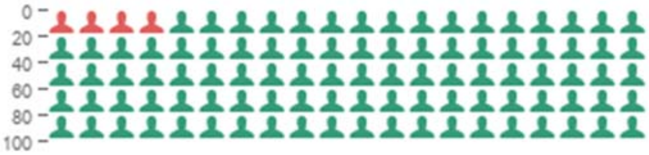

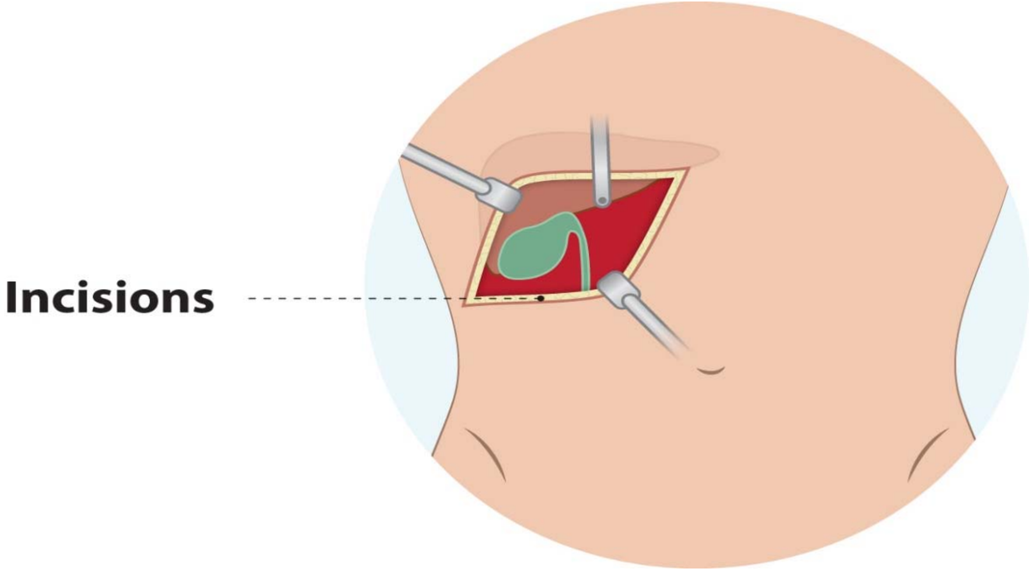

Figure: a bigger incision to remove the gallbladder.

30 out of 100 patients have the same abdominal pain after the operation.

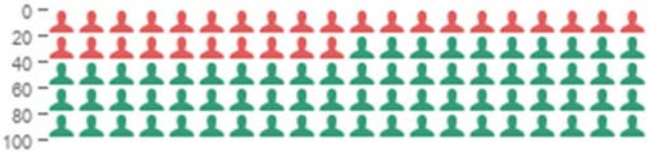

Comparing treatments

	Watchful waiting	Surgery
**What is usually involved?**	You can wait. If you get another attack, you can take painkillers.	During surgery the gallbladder is removed. You can live without gallbladder.
**What are the results?**	33 out of 100 people never have another gallbladder attack.[7]<br><br>You can avoid another attack by exercising and losing weight (not too quickly).	After surgery, 70 out of 100 people will have no more abdominal pain.[6]
**What are the benefits?**	If you have more gallbladder attacks, you can still decide to have surgery.	Best chance of getting rid of your symptoms.
**What are the risks?**	67 out of 100 people will get another gallbladder attack.[7]<br><br>Less than 1 out of 100 people will get an infected gallbladder or pancreas.[3]	30 out of 100 people still suffer abdominal pain after surgery.[6]<br><br>1 out of 100 people get a wound infection.[4]<br><br>1 out of 100 people get an abdominal bleeding.[4]<br><br>In 1 out of 200 people the bile duct gets damaged.[5]<br><br>In 1 out of 500 people the small intestine gets damaged.[4]<br><br>In 4 out of 100 people the incision is extended during surgery. This prolongs recovery after surgery.[4,8]

Key points

Test your knowledge about symptomatic gall stones.

Is surgery always needed in case of gallstones?

- [ ] Yes

In many people their gallstones are asymptomatic. If you suffer gallbladder attacks taking painkillers is an option.

- [ ] No

In many people their gallstones are asymptomatic. If you suffer gallbladder attacks taking painkillers is an option.

After your first gallbladder attack, will you have a second?

- [ ] Yes

1 out of 3 people will not get a new gallbladder attack.

- [ ] No

1 out of 3 people will not get a new gallbladder attack.

Will I be pain free after surgery?

- [ ] Yes

1 out of 3 people still have abdominal pain.

- [ ] No

What is important to you?

Determine what is important for you in choosing treatment for your symptomatic gallstones. Choose the statement that fits you best. Move the orange triangle towards the statement you prefer.

Watchful waiting

I do not have gallbladder attacks very often.

Surgery

I have gallbladder attacks very often.

5	4	3	2	1	0	1	2	3	4	5
Agree					Neutral					Agree

Watchful waiting

I can wait with these complaints.

Surgery

I want to get rid of my complaints as soon as possible.

5	4	3	2	1	0	1	2	3	4	5
Agree					Neutral					Agree

Watchful waiting

I am concerned about the risks of the surgery.

Surgery

I am not concerned about the risks of the surgery.

5	4	3	2	1	0	1	2	3	4	5

Agree Neutral Agree

Your choice

What is your preference?

Watchful waiting

Surgery

5	4	3	2	1	0	1	2	3	4	5

Preference No preference Preference

How certain are you about this decision?

Very uncertain

Very certain

0	1	2	3	4	5	6	7	8	9	10

Very uncertain Very certain

My decision

Please answer the following questions. Is the answer no? Talk to your physician about this.

Knowledge: **Do you know enough about the risks and benefits of each treatment?**

- [ ] Yes
- [ ] No

Preference: **Are you aware of what is most important to you?**

- [ ] Yes
- [ ] No

Support: **Do you feel that you can make the right decision?**

- [ ] Yes
- [ ] No
