# Supplementary material for: The association between patients' preferred treatment after the use of a patient decision aid and their choice of eventual treatment
Source: Health Expect. 2020 Mar 13;23(3):651–8. doi: 10.1111/hex.13045 (PMC7321744; doi:10.1111/hex.13045)
Supplement: Supplementary file 2 — FileS2 [file HEX-23-651-s002.pdf]

Welcome to the PATIENT+ decision aid

How can this decision aid help you?

There are various treatments available for people with a groin hernia. This decision aid provides you with the harms and benefits of each treatment. If you're unsure, your doctor can help you choose the best treatment for you.

At the end you can print this decision aid. This way you can go over it again and discuss everything with people you trust.

What is a groin hernia?

A groin hernia is a bulge in the groin. It occurs when the intestine is pushed through a weak spot in the groin muscle (figure).

This bulge in your groin may be painful or give an unpleasant feeling. However, some people don't have any symptoms at all.[4]

The bulge can slowly increase in size.

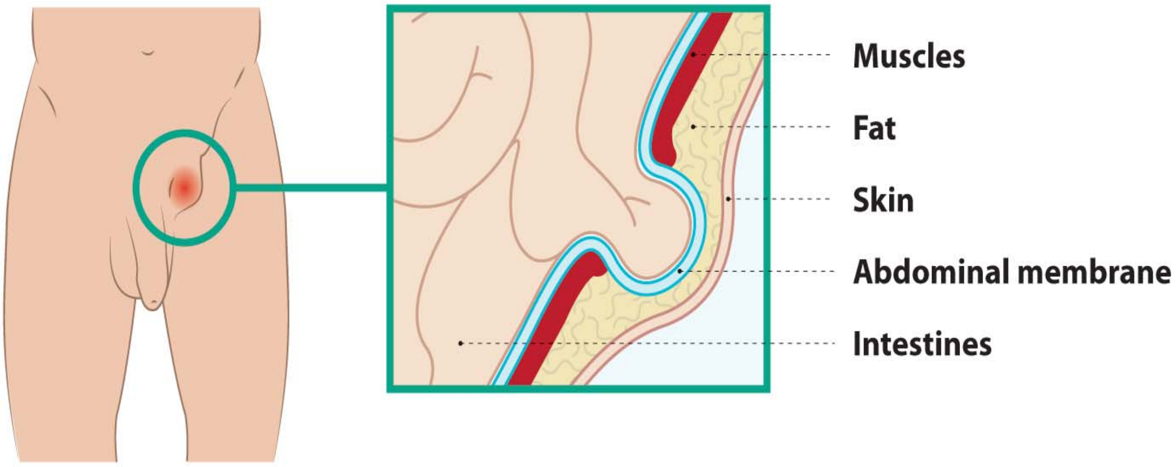

Figure: a groin hernia.

How can a groin hernia be treated?

If your groin hernia is without symptoms, it doesn't need treatment. But a groin hernia won't go away over time. Only surgery can make the bulge go away.

There are two options:

- **Watchful waiting**
- **Surgical repair**

Watchful waiting

Some groin hernias are small and only cause little symptoms. In this case you can decide on watchful waiting.

Other reasons not to have surgery can be:

• You are taking blood thinners and it is not possible to stop them.
• You have other diseases that make an operation too dangerous.
• When you already have a skin infection in your groin area. This increases the risk of wound infection.

If you choose watchful waiting, there is a small chance the hernia becomes incarcerated. When this happens, a piece of intestine gets stuck. The blood flow is blocked and the piece of intestine can die. When the groin hernia becomes incarcerated you suddenly experience a lot of pain. You also start to feel sick. An incarcerated hernia needs immediate surgery. This happens approximately to 3 out of 100 people with a groin hernia.

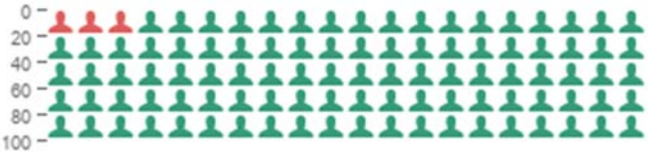

If you choose watchful waiting, and later decide to have surgery, the results of the surgery are the same as surgery right now.

The results of watchful waiting

Because of increasing pain 23 out of 100 people still choose surgery within 2 years.[4,5]

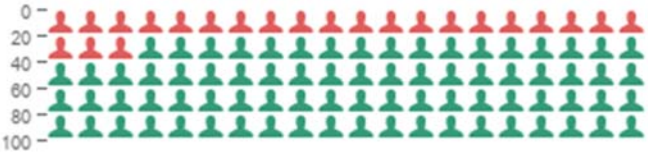

Within 10 years 80 out of 100 people choose surgery.[5]

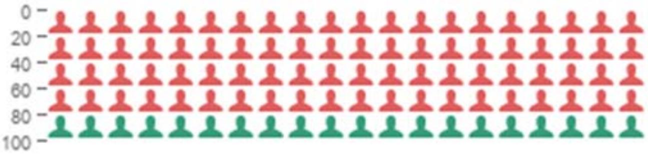

The groin hernia becomes incarcerated in 3 out of 100 people choosing watchful waiting. If this happens, immediate surgery is necessary.[1]

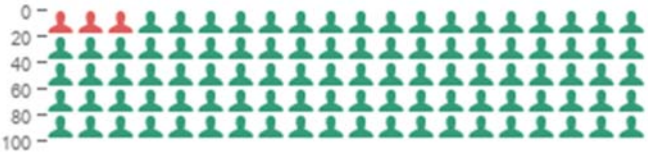

Surgery

During surgery the weak spot in the groin muscle is repaired with a mesh. The mesh ensures that the bulge in the groin won’t return (figure).

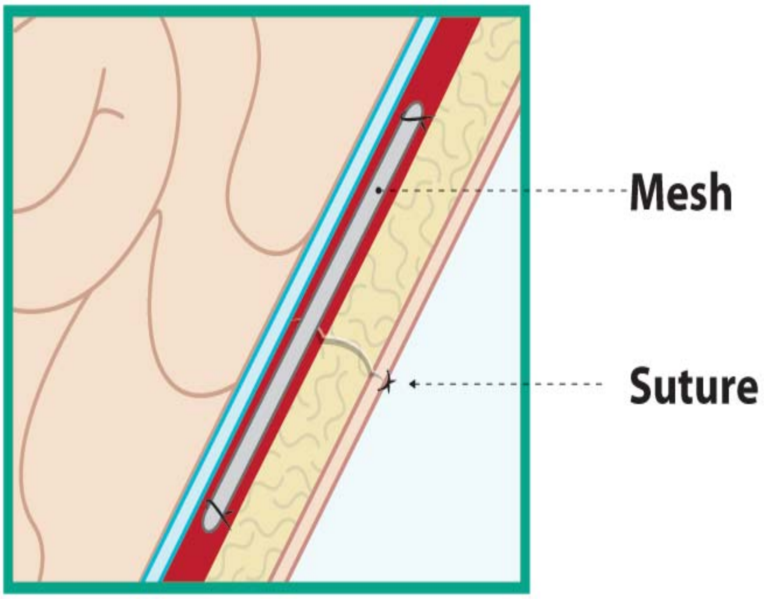

Figure: a mesh to repair the groin muscle.

There are two techniques to repair your hernia.

The first is done through a single incision above the groin (figure).

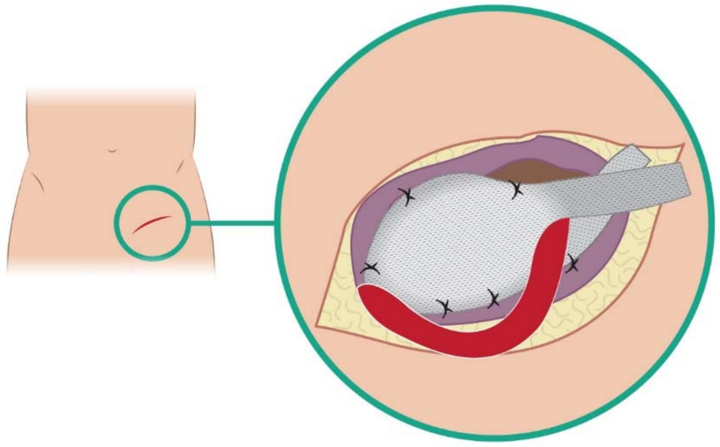

Figure: surgery with one incision.

The second is performed with several small incisions around your abdomen (figure).

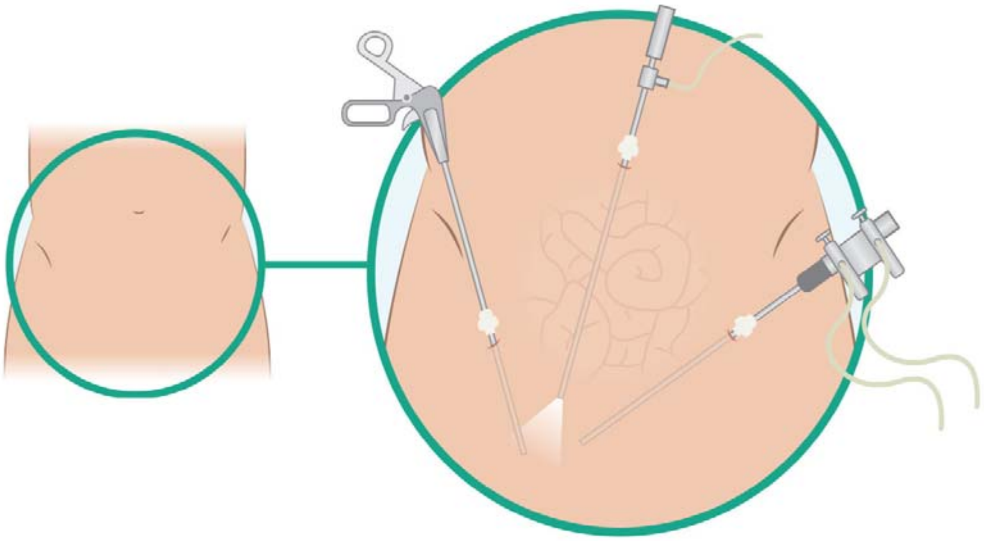

Figure: surgery with several small incisions.

Both techniques have similar results. If you previously had abdominal surgery, the second technique is not available to you. The technique also depends on the surgeon who performs the operation.

The risks of surgery

During or after surgery undesirable things can happen. We call this an adverse event. During groin hernia repair the following can occur:

28 out of 100 people who have a hernia repair have an adverse event.

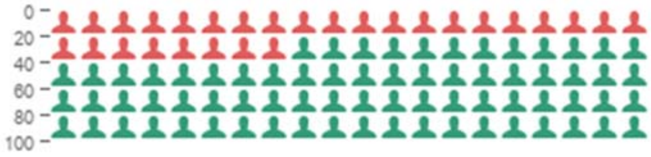

This is only a temporary adverse event in about 27 out of 100 people.

- 15 out of 100 people have a temporary swelling in their groin because of blood or fluid.

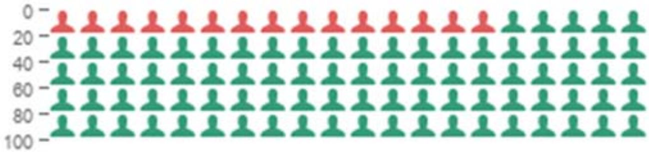

- 3 out of 100 people get a wound infection

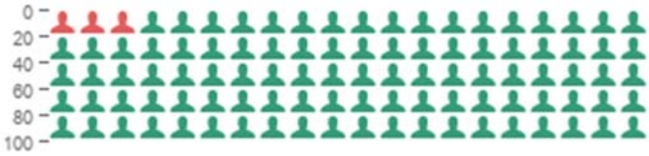

- 10 out of 100 people have groin pain longer than 3 months.[1,2,3,5]

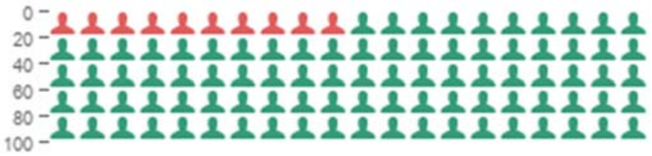

Less than 1 out of 100 people have a severe adverse event:

- Damage to the bladder, intestine or blood vessels.
- In men the sperm duct can be damaged. This decreases fertility.

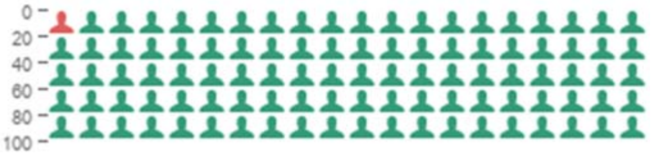

The groin hernia returns in 7 out of 100 people. In this case surgery can be repeated. A second surgery has a higher complication rate.[6]

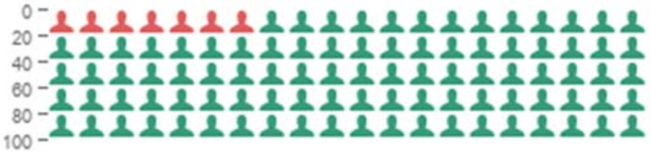

Comparing treatments

	Watchful waiting	Surgery
**What are the advantages?**	No surgery related complications.<br>If you choose watchful waiting now, and later still decide to have surgery, the results of surgery are the same.	After surgery the pain disappears in 90 out of 100 people.<br>No chance of incarceration.
**What are the risks?**	The groin hernia becomes incarcerated in 3 out of 100 people. If this happens immediate surgery is necessary.	28 out of 100 people will have an adverse event.<br>In 27 out of 100 people this is temporary<br>• 15 out of 100 people will have a temporary swelling in their groin because of blood or fluid.<br>• 3 out of 100 people will get a wound infection.<br>• 10 out of 100 people will have groin pain longer than 3 months.[1,2,3,5]<br>Less than 1 out of 100 people have a severe adverse event:<br>• Damage to the bladder, intestine or blood vessels<br>• In men, the sperm duct can be damaged. This decreases fertility.<br>The groin hernia returns in 7 out of 100 people. In this case surgery can be repeated. A second surgery has a higher complication rate. [6]

**How long before the expected result?**	A groin hernia won’t go away over time.<br><br>Because of increasing pain 23 out of 100 people still choose surgery within 2 years	After surgery the groin hernia is gone immediately.

Key points

Test your knowledge about a groin hernia.

Will the groin hernia go over time?

☐ Yes

The groin hernia won’t go away by itself.

☐ No

The groin hernia won’t go away by itself.

Is surgical repair necessary if the bulge is small and causes little symptoms?

☐ Yes

If you have little to no symptoms, surgery is not necessary.

☐ No

If you have little to no symptoms, surgery is not necessary.

Say you will choose watchful waiting now, and later still decide to have surgery. Are the results of the surgery worse?

☐ Yes

People who postpone surgery have similar results later.

☐ No

People who postpone surgery have similar results later.

Do I prevent incarceration of my groin hernia with surgery?

☐ Yes

3 out of 100 people who wait with surgery get an incarcerated groin hernia.

☐ No

What's important to you?

Determine what is important for you in choosing treatment for your groin hernia. Choose the statement that fits you best. Move the orange triangle towards the statement you prefer.

Watchful waiting

The hernia doesn't cause a lot of discomfort.

Surgery

The hernia regularly causes a lot of discomfort.

5	4	3	2	1	0	1	2	3	4	5
Agree					Neutral					Agree

Watchful waiting

I am worried about having surgery.

Surgery

I am not worried about having surgery.

5	4	3	2	1	0	1	2	3	4	5
Agree					Neutral					Agree

Watchful waiting

I am not too worried about the small chance that my groin hernia becomes incarcerated.

Surgery

I am very worried about the small chance that my groin hernia becomes incarcerated.

5	4	3	2	1	0	1	2	3	4	5
Agree					Neutral					Agree

Watchful waiting

I am worried about the risks of surgery.

Surgery

I am not worried about the risks of the surgery.

5	4	3	2	1	0	1	2	3	4	5
Agree					Neutral					Agree

Your choice

What is you preference?

Watchful waiting

Surgery

5	4	3	2	1	0	1	2	3	4	5

Preference

No preference

Preference

How certain are you about this decision?

Very uncertain

Very certain

0	1	2	3	4	5	6	7	8	9	10

Very uncertain

Very certain

My decision

Please answer the following questions. Is the answer no? Talk to your physician about this.

Knowledge: **Do you know enough about the risks and benefits of each treatment?**

- [ ] Yes
- [ ] No

Preference: **Are you aware of what is most important to you?**

- [ ] Yes
- [ ] No

Support: **Do you feel that you can make the right decision?**

- [ ] Yes
- [ ] No
